# Supplementary material for: MicroRNA Profiling as a Predictive Indicator for Time to First Treatment in Chronic Lymphocytic Leukemia: Insights from the O-CLL1 Prospective Study
Source: Noncoding RNA. 2024 Aug 23;10(5):46. doi: 10.3390/ncrna10050046 (PMC11417859; doi:10.3390/ncrna10050046)
Supplement: Supplementary file 1 [file ncrna-10-00046-s001.zip › Supplementary_Nano_et_al/Nano E. et al Figure S1.pdf]

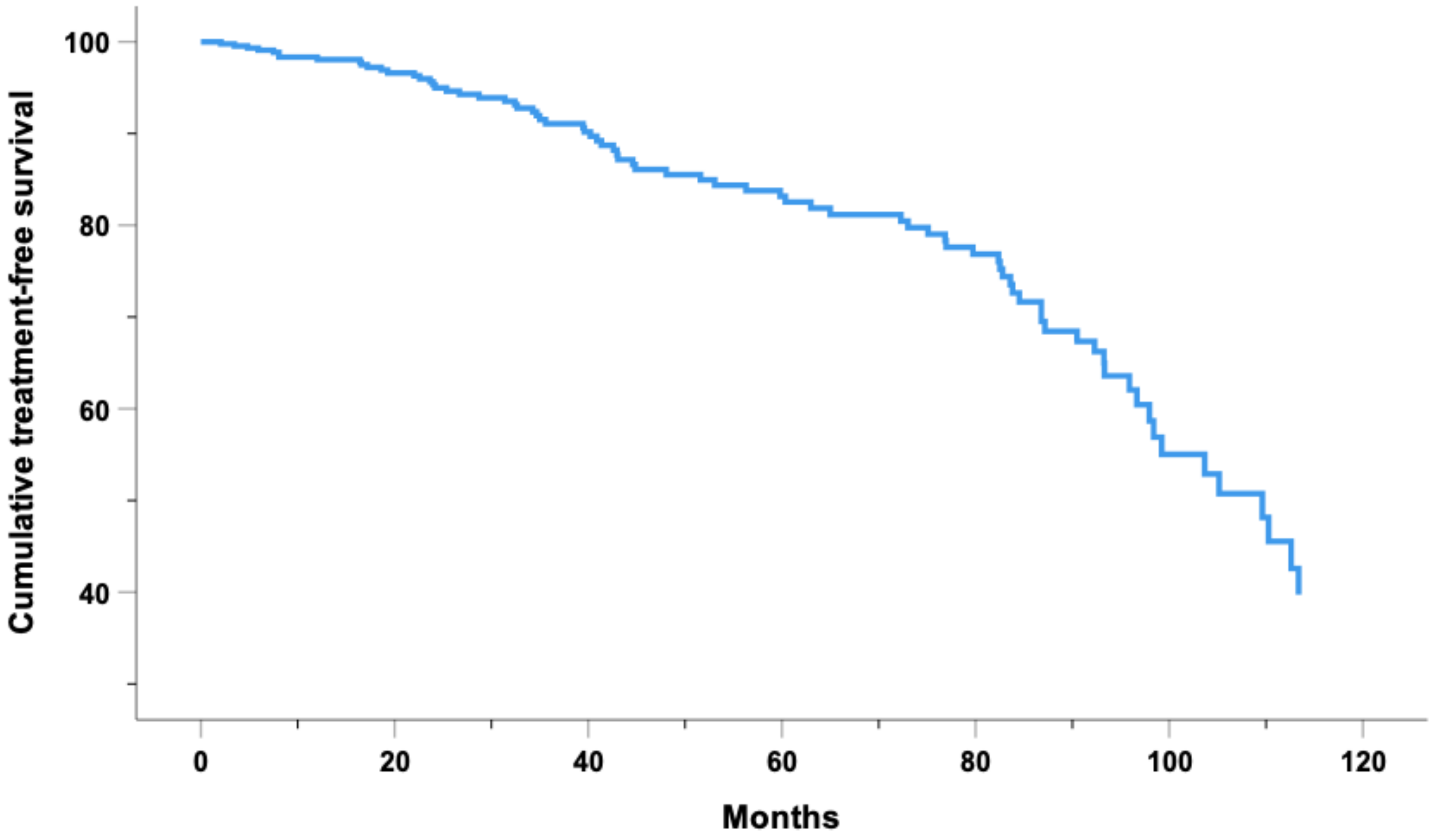

Figure S1. Cumulative treatment-free survival as a function of time. The survival curve was derived from a Cox regression model including Rai stage, β2-microglobulin levels, *IGVH and* NOTCH1 mutational status, del11q, del17p, and the miRNA score (expanded model), with all these variables set to the corresponding mean values.
