# Supplementary material for: MicroRNA Profiling as a Predictive Indicator for Time to First Treatment in Chronic Lymphocytic Leukemia: Insights from the O-CLL1 Prospective Study
Source: Noncoding RNA. 2024 Aug 23;10(5):46. doi: 10.3390/ncrna10050046 (PMC11417859; doi:10.3390/ncrna10050046)
Supplement: Supplementary file 1 [file ncrna-10-00046-s001.zip › Supplementary_Nano_et_al/Nano E. et al Table_S2_Nano_et_al.pdf]

Table S2: Evidence of interactions between genes and miRNAs shown in figures 3 and 4 according to mirTarbase, MicroCosm, and TargetScan

Each column represents evidences supporting a gene - miRNA interaction for mirTarbase, or if it is reported by MicroCosm or Targetscan.

			miRTarBase										microCosm_v5	targetScan_v6.2
			Validation methods								Sum	# of papers		
			Strong evidence			Less strong evidence								
Species (Target)	miRNA	Target	Reporter assay	Western blot	qPCR	Microarray	NGS	pSILAC	Other	CLIP-Seq				
Homo sapiens	hsa-miR-29c-3p	KDM5B					X				1	1		X
		ANKRD52												X
		ZBTB34												X
		UBFD1												X
		ABHD4												X
		FBXL20												X
		PRICKLE1											X	
		H2AFY2											X	
		MRPS26											X	
Homo sapiens	hsa-miR-33a-3p	IGF1R					X			X	2	8		
		ANKRD52					X			X	2	2		
Homo sapiens	hsa-miR-625-5p	IGF1R	X	X	X		X			X	5	8		
		ALDH16A1											X	
Homo sapiens	hsa-miR-150-5p	GNE				X				X	2	2		
		IGF1R	X	X		X					3	0		
		ZNF467											X	
		SAMD4B											X	
		ANKRD52												X
		UBFD1												X
Homo sapiens	hsa-miR-296-3p	ANKRD52					X			X	2	2		
		DCAF8					X				1	1		
		KDM5B					X				1	1		
		MLEC					X				1	1		
		CHD3											X	
		ALDH16A1											X	
Homo sapiens	hsa-miR-144-5p	LBR					X			X	2	2		
Homo sapiens	hsa-miR-148a-3p	LBR					X				1	1	X	X
		MLEC					X			X	2	4		X
		ZNF490					X				1	1		
		IL17RA											X	
		ABI3											X	
		CHD1L											X	
		ANKRD52												X
		PI4KA												X
		UBFD1												X
		CREB3L2												X
		CUL5												X
Homo sapiens	hsa-miR-193a-3p	CIAO1					X				1	1		
		UBFD1					X				1	1		
Homo sapiens	hsa-miR-28-5p	PROSER3								X	1	3		
		TSC22D1					X			X	2	2		
		CDCA2											X	
		CREB3L2											X	
		FIBP											X	
		ANKRD52												X
Homo sapiens	hsa-miR-502-5p	IGF1R					X			X	2	6		X
		H2AFY2											X	
		IGF1R												X
		CUL5												X
Homo sapiens	hsa-miR-1-3p	FADD				X					1	1		
		HOOK1				X					1	1		X
		SLC39A6											X	
		PIGP											X	
		YLPM1											X	
		CREB3L2											X	
		GNE												X
		MKL1												X
		FBXL20												X
Homo sapiens	hsa-miR-99a-5p	IGF1R	X	X	X				X		4	6		X
Homo sapiens	hsa-miR-124-3p	CREB3L2				X			X		2	1		X
		MLEC				X					1	1		X
		ABHD4				X					1	1		X
		H2AFY2											X	
		TSPO											X	
		FBXL20											X	
		TSC22D1											X	
		APBB2												X
		ZBTB34												X
		SAMD4B												X
		CUL5												X
Homo sapiens	hsa-miR-516a-5p	CHD1L											X	
Homo sapiens	hsa-miR-582-3p	SLC39A6											X	X
		IL17RA												X
Homo sapiens	hsa-miR-671-5p	MRPS26											X	
		ANKRD52												X
		CREB3L2												X
