# Supplementary material for: MicroRNA Profiling as a Predictive Indicator for Time to First Treatment in Chronic Lymphocytic Leukemia: Insights from the O-CLL1 Prospective Study
Source: Noncoding RNA. 2024 Aug 23;10(5):46. doi: 10.3390/ncrna10050046 (PMC11417859; doi:10.3390/ncrna10050046)
Supplement: Supplementary file 1 [file ncrna-10-00046-s001.zip › Supplementary_Nano_et_al/Nano E. et al Table S1.pdf]

Table S1. Univariable (A) and Multivariable Cox Regression (B) analyses for TTFT with identification of significant Independent Predictors via backward elimination strategy.

**Univariable analysis (A)**		**95% CI**		
**Variables**	**HR**	**Lower limit**	**Upper limit**	**P-value**
***IGHV*, unmutated**	4.708	3.085	7.185	<0.001
**ZAP-70, positive**	2.914	1.914	4.434	<0.001
**del11q**	6.341	3.554	11.315	<0.001
**del17p**	4.076	1.63	10.191	0.003
**NOTCH1, mutated**	2.112	1.272	3.507	0.004
**B2M, abnormal**	2.23	1.42	3.503	<0.001
**Rai stage, I-II**	1.782	1.157	2.747	0.009
**Lymphocytosis, >5x10^9/L**	1.806	1.005	3.245	0.048
**CD38, positive**	2.692	1.754	4.134	<0.001
**Multivariable Cox regression analyses (B)**				
***IGHV*, unmutated**	3.452	2.273	5.244	<0.001
**del11q**	2.871	1.556	5.299	<0.001
**del17p**	9.616	2.212	41.813	0.003
**NOTCH1, mutated**	1.866	1.129	3.086	0.015
**B2M, abnormal**	1.940	1.351	2.785	<0.001
**Rai stage, I-II**	2.117	1.443	3.107	<0.001
